# Progress in Cerebral, Spinal, and Nerve Surgery

**Published:** 1904-03-26

**Authors:** 


### Progress in Cerebral, Spinal, and Nerve Surgery.

(*Concluded from page* 449.)

**A Case of Excision of the Gasserian Ganglion for Trigeminal Neuralgia.**—Gordon[4] reports this case. The patient was a woman æt. 63. Her first attack of neuralgia came on suddenly eight years ago. It began on the left side of the upper lip and cheek, and spread to the temple. The pain was as if a knitting-needle had been bored into the left temple and turned round. Ever since this the attacks have recurred with varying frequency. At present the attacks are very violent, come on about every ten minutes, and last a minute or less. The paroxysms are worse at night, giving rise to fearful nightmares. The pain at one time caused her to attempt suicide. All the branches of the left fifth nerve except the lingual were involved from time to time. Tender points were present in different places. All the teeth had been extracted without relief. The patient's condition being a truly pitiable one. Drug treatment having proved useless, and temporary relief only likely to be obtained from neurectomy, it was decided to remove the Gasserian ganglion. The Horsley-Krause operation was selected. The writer divides it into four stages. *First stage*: a large horseshoe flap, including a large piece of temporal muscle, is turned down, the skull opened with a medium-sized trephine, and the opening enlarged to the required size by bone forceps. This the writer considers better than turning down an osteo-plastic flap, owing to the time the latter takes to cut. The canal for the middle meningeal was opened and had to be plugged with aseptic wax. *Second stage*: the dura is raised from the middle fossa as far as the points of exit of the two lower divisions of the nerve. Two points of fixation of the dura constitute important guides. During this stage of the operation hæmorrhage is often very troublesome, sometimes necessitating postponement of the subsequent steps of the operation. This can generally be avoided by plugging the foramina ovale and spinosum with pledgets of gauze. *Third stage*: the opening up of Meckel's space and the complete exposure of the nerves and ganglion. This is done by incising the dura just where the nerves are leaving the skull. *Fourth stage*: the nerves are divided close to their points of exit from the skull and dissected up to the ganglion, and the latter removed by evulsion. The sensory root will probably tear away from the side of the pons. The proximity of the cavernous sinus must ever be remembered. If it is wounded, however, the hæmorrhage is readily controlled by pressure. The operation is sometimes followed by aphasia or ocular palsies, but these usually have been transient only. The writer's case had a temporary partial third-nerve paralysis. Eight months after the operation she was in good health and entirely free from her neuralgia. Considering the serious nature of the operation the mortality is low; Horsley in 1901 having had two deaths in 22 operations, and Krause three in 25.

**Intraneural Injection of Osmic Acid for Supra-**

orbital Tic.—Abbe[5] showed a patient that he had operated on by this method. When first shown he was suffering from a severe form of facial tic limited to the supra-orbital branch of the fifth nerve. The operation was performed under cocaine anæsthesia, an incision being made beneath the eyebrow, and the nerve isolated at the supra-orbital foramen. The nerve was elevated and thoroughly infiltrated with a 1½ per cent. solution of osmic acid, the excess being immediately wiped away. The operation caused very little pain. On the day following, the painful tic occurred only 15 times as compared with 40 to 60 shocks daily before operation, and it diminished each day until it had quite ceased by the fifth day, and the patient had remained well since (a month). Some anæsthesia of the scalp resulted from the operation, but the cornea and conjunctiva were not affected. Dr. Abbe could not say whether the relief would be permanent.

**Ischæmic Paralysis.**[6]—This condition is one of paralysis, with contraction of the fingers and wrist, which comes on more or less rapidly after a severe injury to the elbow or forearm, generally in children. There is great deformity and crippling of the hand, the condition if untreated becoming progressively worse; but a good result may be hoped for if the treatment is carried out resolutely for a very long period. The essential factor in the production of the contracture is the series of changes set up in the muscles of the forearm, the circulation through which has been intercepted by severe or long-continued pressure by splints or the compression of bandages. These changes are, briefly, the conversion of the muscle fibres into fibrous tissue. In the majority of cases there is no anæsthesia or other evidence of nerve injury. With the symptoms mentioned above there is usually intense swelling of the forearm and discoloration of the fingers, and an absence of pain. A typical deformity is produced. On extending the wrist, the terminal phalanges of the fingers and the last joint of the thumb are firmly flexed into the palm—so firmly that it is impossible to straighten the fingers until the wrist is fully flexed. Later the forearm becomes hard and wasted, the wrist semi-flexed and all movements much limited. The diagnosis in a typical case is made in a child who has had a severe injury of the forearm or elbow. Splints have been applied, the arm has become intensely swollen, and paralysis sets in. The onset of paralysis and contracture is simultaneous and painless. The electrical reactions are normal and the typical deformity is present. Injuries of the ulnar, median, and musculo-spiral nerves are accompanied by definite types of paralysis and areas of anæsthesia. With regard to the pathology of the condition, Volkmann's explanation was that rigor mortis set in in the muscles, causing the paralysis and rigidity. While a true myositis is recognised by most observers, this has been variously ascribed to the ischæmia alone; to the rapid effusion of fibrous-tissue-forming element; as secondary to the inflammatory process of the pressure-sores or sloughs of skin; or to cellulitis spreading up the forearm from a whitlow or septic wound; or to actual laceration of the muscles. At operation the muscles have been found wasted, dry, pale, and fibrous; the nerves embedded in fibrous tissue, with swollen sheaths. The prognosis will depend on the care and patience with which the treatment is carried out, and the results of the latter will of course largely depend on the amount of muscle that remains to represent the whole. The use of electricity, hot-air baths, douches, and especially massage, and passive movement, form the basis of the treatment. Operative measures, in the direction of tenotomy or of tendon-lengthening, may be called for. Whatever treatment is adopted, it must be persevered with in a most systematic and persistent manner.

[4] Dublin Jour. of Med. Sci., Oct. 1, 1903. [5] Med. Rec., Jan. 23, 1904. [6] Birm. Med. Rev., Oct. 1903.

